# LPS-induced Vitamin D Receptor Decrease in Oral Keratinocytes Is Associated With Oral Lichen Planus

**DOI:** 10.1038/s41598-018-19234-z

**Published:** 2018-01-15

**Authors:** Bin Zhao, Ran Li, Fang Yang, Feiyan Yu, Na Xu, Fang Zhang, Xuejun Ge, Jie Du

**Affiliations:** Department of Stomatology, Shanxi Medical University, Taiyuan, Shanxi China

## Abstract

The suppressive function of vitamin D on oral lichen planus (OLP) have been documented previously. Vitamin D receptor (VDR) expression is down-regulated in OLP, but the molecular mechanism of its decrease and the related anti-inflammatory contributor of epithelial VDR signaling is unclear. Herein, we demonstrated that lipopolysaccharide (LPS) remarkedly down-regulated VDR expression of keratinocytes, and the reduced regulation was dependent on tumor necrosis factor alpha (TNFα)-miR-346 pathway. In human specimen studies, VDR levels of oral mucosal epithelia from OLP patients decreased substantially accompanied with robust TNFα and miR-346 induction, compared to the normal tissues. In addition, vitamin D/VDR signaling inhibited LPS-induced p53-upregulated modulator of apoptosis (PUMA) induction in keratinocytes via impeding nuclear factor-κB (NF-κB) activation, resulting in keratinocytes apoptosis reduction. Importantly, PUMA activity was up-regulated strongly in diseased epithelium, reversely correlated with VDR expression. Totally, our data indicate that LPS is responsible for VDR downregulation in oral keratinocytes, which is associated with OLP development.

## Introduction

Oral Lichen Planus (OLP) is recognized as a chronic inflammatory disease featured with a T-cell infiltrated band in the lamina propria^1,2^. Albeit the pathogenesis and etiology of this disorder are still elusive, it is clear that derangements in these complex elements, such as environmental, microbial dysbiosis, autoimmune and heredity factors, initiate OLP and exacerbate the development of it^3^. Patients with OLP mostly suffer from clinical feature regarding burning mouth, even in the process of eating^3,4^. In addition, OLP is considered to possess the character of precancerosis, which has been confirmed by numerous studies^5,6^. Among the six established patterns of OLP (reticular, plaque, papular, atrophic, bullous and erosive subtypes), erosive-form lesion is believed to be the most threatening and takes the highest risk of canceration^7^. Given its clinical uncomfortable symptom and potential of malignant transformation, exploration concerning exact pathogenic contributor and targeted agent seems urgent in the field of OLP.

Previous studies have provided compelling evidence that CD4^+^ and CD8^+^ T cells, trigged by multiple intrinsic or extrinsic etiological factors, are the major mediators in the inflammatory response of OLP^8–10^. What is more, CD8^+^ T cells as well as mast and dendritic cells are referred to disrupt the physical integrity of epithelium by damaging epithelial cells mainly^11^. Additionally, other studies suggest oral bacteria would break down the epithelial barrier of mucosa, enhance harmful substances penetration and induce T cells infiltration^12^. Indeed, a plentiful of bacteria are demonstrated to exist both within the lamina propria and in epithelial layer, positively correlated with the status of infiltrated T cells^12^. The histological characteristics of disrupted epithelium of OLP caused by bacteria or inflammatory reaction, such as apoptosis, atropy and liquefaction, indicate mucosal epithelial barrier impairment^13^. It is worth noting that increased epithelial cells apoptosis which has been reported in the lesions of OLP patients results in physical mucosal barrier breakdown, epithelial layer thickness reduction and homeostasis dysfunction^14,15^. Inflammatory reaction causes apoptosis of epithelia^16^, in turn, excessive loss of these cells accelerates invasion of oral bacteria and antigen. This vicious circle leads to the clinical manifestations of OLP finally.

1,25- dihydroxyvitamin D (1,25(OH)_2_D_3_), the active form of vitamin D (VD), is recognized as a pleiotropic hormone and possesses comprehensive physiological activities^17^. The biological function of 1,25(OH)_2_D_3_ is mostly regulated by its specialized nuclear hormone receptor, vitamin D receptor (VDR), which significantly expressed in epithelial cells of diverse tissues^18^. The deficiency of vitamin D is closely related with enhanced risk of inflammatory diseases, such as OLP and inflammatory bowel disease^19,20^. Our previous studies have noted that insufficiency of serum 25-hydroxyvitamin D (25(OH)D) is detected in patients with established OLP. Consistently, VDR levels in the diseased mucosal tissues of OLP are almost 50% reduced, accompanied with immunoreactivity induction^20^. Despite we also confirmed that vitamin D plays its protective role in OLP through mediating nuclear factor-κB (NF-κB) signaling pathway, the function of epithelial VDR of oral mucosa remains elusive, requiring more investigations. Since intestinal epithelial VDR signaling maintains the integrity of gut mucosal barrier dependent on inhibiting cell apoptosis and regulating tight junction of epithelium^19,21^, we propose that VDR located in oral epithelium might preserve mucosal homeostasis as well. Local VDR reduction in epithelium, due to inflammation-induced chemokines or lipopolysaccharide (LPS)-induced cytokines partially, might compromise epithelial protective function and accelerate the onset of OLP.

Herein, we aimed to explain the molecular mechanism of epithelial VDR reduction in OLP and explore how VD-VDR signaling of oral epithelium inhibits the initiation or development of OLP. Our findings show that epithelial VDR decrease is driven by LPS-induced miR-346, exaggerating epithelial cell apoptosis through inducing pro-apoptotic factor p53-upregulated modulator of apoptosis (PUMA) further.

## Methods and Materials

### Human biopsies

Buccal mucosal biopsies were collected from OLP patients at the stomatological hospital of Shanxi Medical University. Samples of patients were got from diseased and adjacent normal mucosa by histopathological examination. Human specimens were digested by 0.25% dispase II for 12 hours at 4 °C. Epithelium and lamina propria were separated using muscle forceps as described^10^. OLP identification and patient inclusion criteria were in term of the modified WHO diagnostic criteria^22^ and previous studies^23^, respectively. Clinical data of OLP patients in this investigation were provided in Table 1. Informed consent with signature was obtained from each individual involved in this work. All procedures associated with human studies were under the approval from the Institutional Ethical Committee of Shanxi Medical University (Protocol # 2016 LL046). All experiments were performed in accordance with relevant guidelines and regulations.

**Table 1:** Clinical parameters of OLP patients involved in this study.

NO.	Age	Gender	Sites	Subtypes
1	42	F	Buccal mucosa	Reticular
2	32	M	Buccal mucosa	Reticular
3	51	F	Buccal mucosa	Reticular
4	31	F	Buccal mucosa	Reticular
5	46	F	Buccal mucosa, Tongue	Reticular, Plaque
6	28	M	Buccal mucosa	Reticular
7	59	F	Buccal mucosa	Reticular
8	47	F	Buccal mucosa, Tongue	Reticular, Plaque
9	34	F	Buccal mucosa	Reticular
10	65	F	Buccal mucosa	Reticular
11	46	F	Buccal mucosa, Tongue	Reticular, Plaque
12	52	F	Buccal mucosa	Reticular
13	59	M	Buccal mucosa	Reticular
14	66	M	Buccal mucosa	Reticular

### Cell culture

HaCat cells (from ATCC) used in this study were cultured with dulbecco minimum essential medium (DMEM) containing 10% fetal bovine serum (FBS) and 1% P/S, under 37 °C and 5% CO_2_ condition. For mimicking the microenvironmental status of OLP, HaCat cells were cultured in keratinocyte serum-free medium (KSFM) overnight as previously described^10^. Cells were pre-treated with tumor necrosis factor alpha (TNFα) neutralizing antibodies (4 µg/ml, R&D Systems) for 12 hours, followed by 24-hour E.coli LPS (100 ng/ml) stimulations. In separate experiments, cells were stimulated with TNF (100 ng/ml) for 24 hours after a 24-hour transfected treatment of miR-346-specific hairpin inhibitor. As detailed^24^, HaCat cells were transfected with microRNA oligo mimics or constructed plasmids with Lipofectamine 2000 (Life Technologies). LPS, TNF and miRNA inhibitor were from Sigma-Aldrich (St. Louis, MO, USA). 1,25(OH)_2_D_3_ and plasmids were provided by Dr. Li YC (The University of Chicago).

### Western blot

HaCat cells and epithelia of biopsies were denatured with Laemmli buffer, centrifuged and undergone a 10-min heat at 95 °C. Proteins in lysates were separated by SDS-PAGE, followed by electrophoretically transferred onto Immobilon-P membranes (Millipore, USA). Analyses of western blot were completed as described^25^. A series of primary antibodies were utilized, anti-TNFα, anti-VDR and anti-β-actin from Santa Cruz (California, USA); anti-PUMA, anti-caspase 3, anti-caspase 9, anti-Bcl2, anti-Bax, anti-Bak, anti-p53, anti-phospho-NF-κB p65, anti-NF-κB p65 from Cell Signaling (Beverly, MA).

### Real-time PCR

TRIzol reagent (Invitrogen, USA) was used to isolate total RNAs. Real-time PCR was operated in a real-time system (Bio-RAD IQ5) with SYBR Premix Ex kit (TaKaRa, Japan). Relative amount of transcripts was calculated by the 2^−ΔΔCt^ formula. PCR primers are displayed in Table 2.

**Table 2:** Primer sequences used in this work.

Primer name	Forward (5′-3′)	Revere (5′-3′)
hsa-mir-346	TGTCTGCCCGCATGCCTGCCTCT	
PUMA κB ChIP	CATGTAAGTGATGTCATATGTC	CTTCCTGGTCTTTTCCAAACT

### Luciferase reporter assays

Luciferase reporter and hsa-miR-346 oligo mimic or plasmids were co-transfected into HaCat cells by Lipofectamine 2000 in every single experiment. Cells were stimulated by LPS or 1,25(OH)_2_D_3_ in transfected assays, and then lysed after 24-hour treatments. Luciferase activity was measured by the Dual-Luciferase Reporter assay kit (Promega) as mentioned before^24^.

### Histology and immunostaining

Freshly collected buccal biopsies were fixed for 24 hours with 10% formalin in phosphate buffer saline (PBS) (pH 7.4) as described^19^. In immunostaining assay, slides were treated by anti-VDR or anti-PUMA antibodies at first, and then incubated with horse radish peroxidase (HRP)-conjugated secondary antibodies. Slides were observed using an Olympus IX51 fluorescence microscope.

### Chromatin Immunoprecipitation Assays

Chromatin immunoprecipitation (ChIP) assays were carried out with anti-p65 antibodies as reported previously^26^. Real-time PCR was used to quantify the assays. Primers^21^ were manifested in Table 2.

### Statistical analysis

All data values were presented as means ± standard deviation (SD). Statistical comparisons were performed using unpaired two-tailed Student’s *t*-test or one-way analysis of variance (ANOVA) as appropriate. P < 0.05 was considered statistically significant.

## Results

### VDR expression is down-regulated by LPS accompanied with TNFα up-regulation

**Figure 1:**
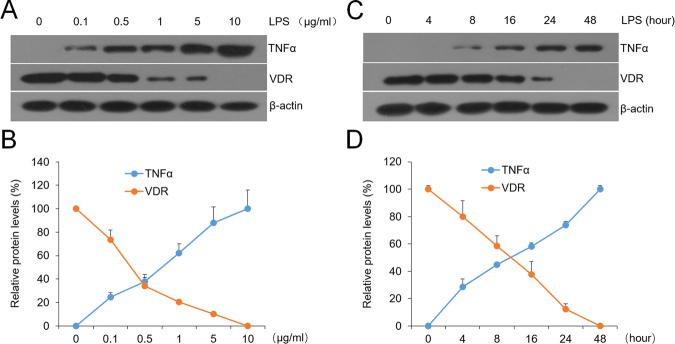
VDR is largely down-regulated by LPS in HaCat cells. (**A**) Western blot measurements of HaCat cells treated with concentration-dependent LPS or saline for 24 hours using different antibodies as indicated. (**B**) Relative protein levels of TNFα and VDR with different doses as shown. (**C**) Western blot measurements of HaCat cells treated with time course-dependent LPS (10 µg/ml) or saline. (**D**) Relative protein levels of TNFα and VDR with different treatment times as noted.

Oral bacteria invasion is thought to be one of the etiopathogenic contributors of OLP, while LPS, one component of the cell-wall of bacteria, is believed to be a potent endotoxin which can trigger a pattern of inflammatory reaction in mucosa^10^. To verify the hypothesis that LPS reduces VDR expression, we assessed the VDR levels in HaCat cells in the presence of LPS. As shown in Fig. 1, VDR expressions were reduced in a concentration-dependent manner after LPS treatment, while TNFα levels were augmented inversely (Fig. 1A and B). Consistent with these results, time course experiments also exhibited VDR decrease and TNFα increase following LPS (10 µg/ml) stimulation (Fig. 1C and D). These observations suggest that LPS can reduce VDR expression *in vitro*.

### LPS-induced decrease of VDR is mediated by TNF-dependent miR-346

**Figure 2:**
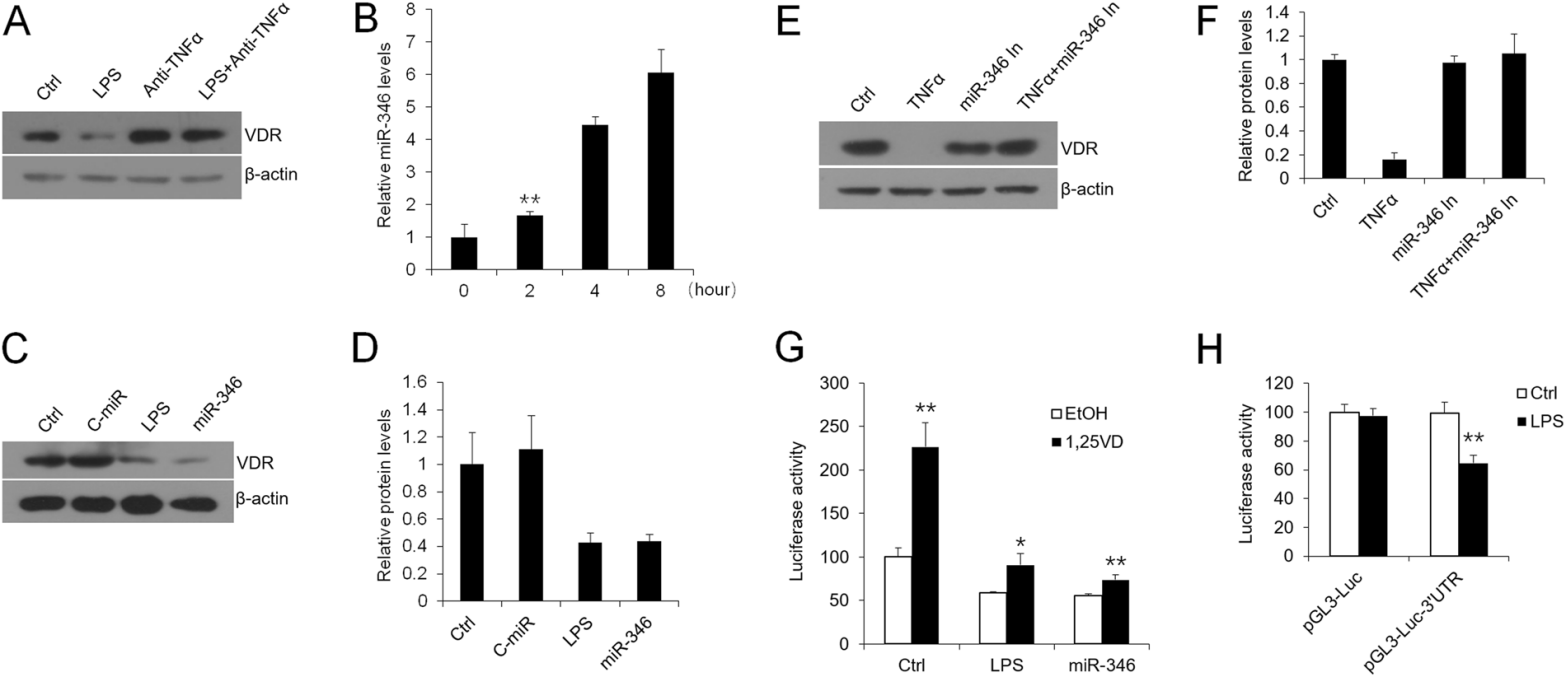
TNFα-dependent LPS suppresses VDR expression via miR-346 regulation. (**A**) Western blot measurements of HaCat cells treated or untreated with LPS in the presence or absence of anti-TNFα. (**B**) Real-time PCR quantitation of miR-346 treated by TNFα with different times, **P < 0.01 vs. Ctrl. (**C** and **D**) Western blot measurements of HaCat cells treated with control miRNA, LPS or miR-346 (**C**) and quantitative analyses (**D**). (**E**) Western blot measurements of HaCat cells treated or untreated with TNFα in the presence or absence of miR-346 inhibitor. (**F**) Relative protein levels of VDR with different treatments as indicated. (**G**) HaCat cells were co-transfected with p3xVDRE-Luc and control miRNA, LPS and miR-346 as shown. 24 hours later, the cells were treated with ethanol (ETOH) or 1,25(OH)_2_D_3_ (20 nM) and then tested by luciferase activity assay, *P < 0.05, **P < 0.01 vs. corresponding Ctrl. (H) HaCat cells were transfected with pGL3-Luc or pGL3-Luc-3′UTR, followed by 12-hour LPS (10 µg/ml) or saline stimulations, **P < 0.01 vs. corresponding Ctrl. Ctrl, control; 1,25VD, 1,25(OH)_2_D_3_.

To investigate the effect of TNFα which is known to be stimulated by LPS, we measured VDR expression in the absence of its production in HaCat cells. As shown in Fig. 2, LPS-stimulated decrease of VDR levels was blocked by anti-TNFα neutralizing antibody (Fig. 2A). This finding demonstrates that LPS down regulates VDR expression in a TNFα-dependent way. Furthermore, TNFα-induced miR-346 is recognized to target VDR directly^24^. We then asked whether miR-346 modulates VDR expressions in oral epithelial cells. As expected, levels of miR-346 were substantially up-regulated by TNFα in keratinocytes (Fig. 2B). At the same time, miR-346 attenuated the expression of VDR by targets it compared with that of LPS which served as a positive control (Fig. 2C and D), and miR-346 inhibitor counteracted the decrease of VDR in the presence of TNFα (Fig. 2E and F).

To investigate whether miR-346 embraces the ability of suppressing the VDR’s transactivating activity, we transfected p3xVDR element (VDRE)-Luc reporter plasmid into HaCat cells with control miRNA oligo mimic, LPS and hsa-miR-346 oligo mimic in the presence or absence of 1,25(OH)_2_D_3_. As shown, miR-346 and LPS markedly impeded 1,25(OH)_2_D_3_-induced luciferase activity in keratinocytes (Fig. 2G), reflecting miR-346 did block the transactivating activity of VDR. Furthermore, LPS suppressed VDR through targeting the site in the 3′ untranslated region (3′UTR) (Fig. 2H). The construction of pGL3-Luc-3′UTR plasmid has been described previously^24^.

### VDR decrease in OLP patient biopsies is correlated with increase of TNFα and miR-346

**Figure 3:**
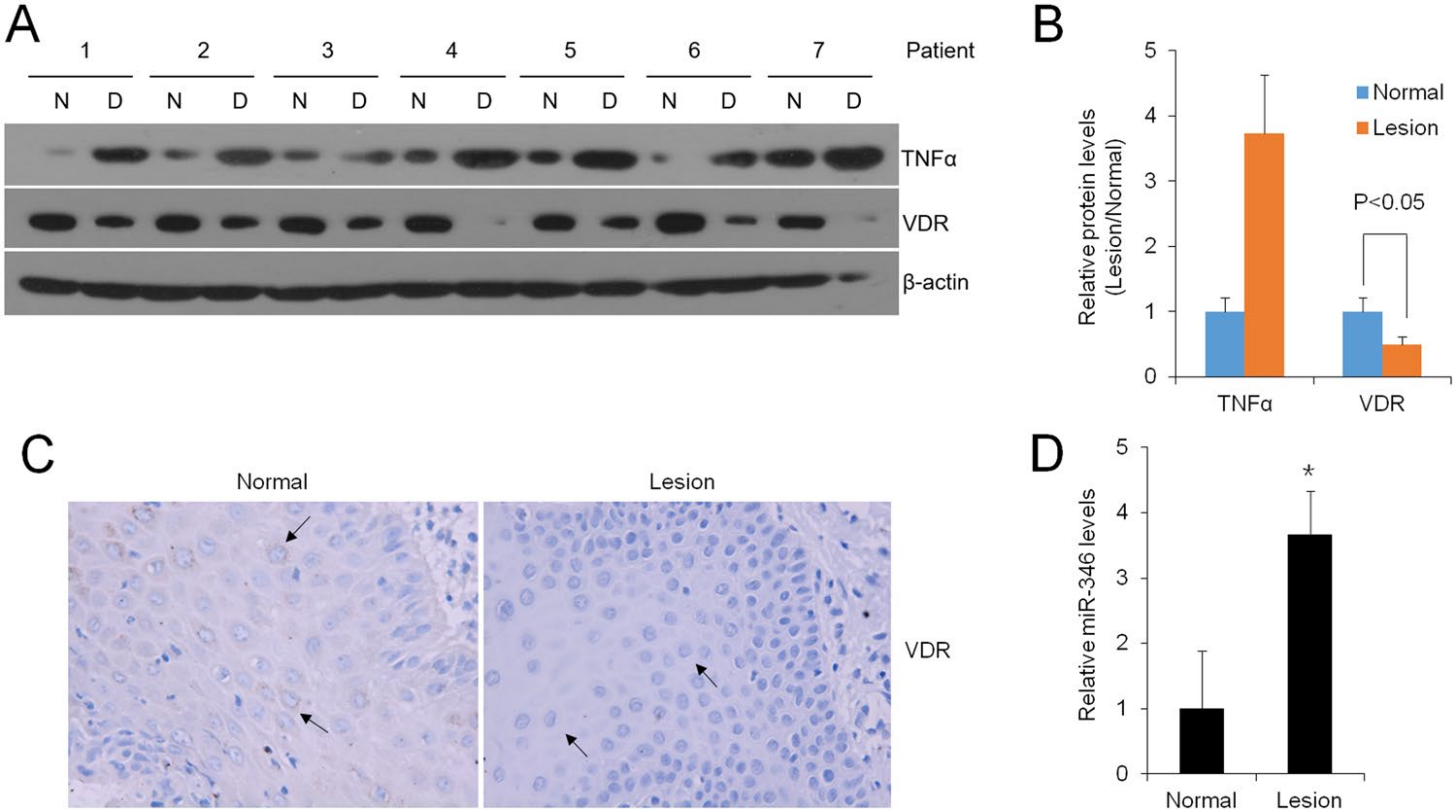
VDR decrease is significant in buccal mucosal biopsies of OLP patients. (**A**) Western blot measurements of lesion tissues (L) and adjacent normal biopsies (N) from OLP patients. (**B**) Relative protein levels of TNFα and VDR in the diseased tissues in contrast to normal ones analyzed by densitometry, n = 14. (**C**) Immunostaining with anti-VDR antibody in buccal mucosal biopsies. Magnification 400× . (**D**) Relative mRNA levels of miR-346 in biopsies quantified by Real-time PCR, *P < 0.05, vs. control, n = 14.

Our previous observations indicated that oral mucosal VDR status of OLP patients was down-regulated by 50%^20^. Here, to address the function of VDR in oral epithelial cells, we isolated the epithelial layer of oral mucosal tissues from a new cohort of patients suffered from OLP. As displayed in Fig. 3, compared to the adjacent normal samples, levels of VDR protein were almost considerably reduced in the diseased epithelium (Fig. 3A and B). Importantly, the VDR decrease was related with dramatic and robust increase of TNFα (by 380%) in the inflamed biopsies (Fig. 3A and B). Meanwhile, immunostaining data also exhibited that the VDR levels in the lesion were much lower in contrast to the normal ones (Fig. 3C). As expected, levels of miR-346 in diseased regions were up-regulated by 350%, evaluated by qPCR (Fig. 3D). Thus, VDR expression is reversely connected with TNFα activity and miR-346 in human patients, consistent with the results *in vitro* mentioned above.

### 1,25(OH)_2_D_3_ suppresses LPS-induced PUMA activation by blocking NF-κB pathway

**Figure 4:**
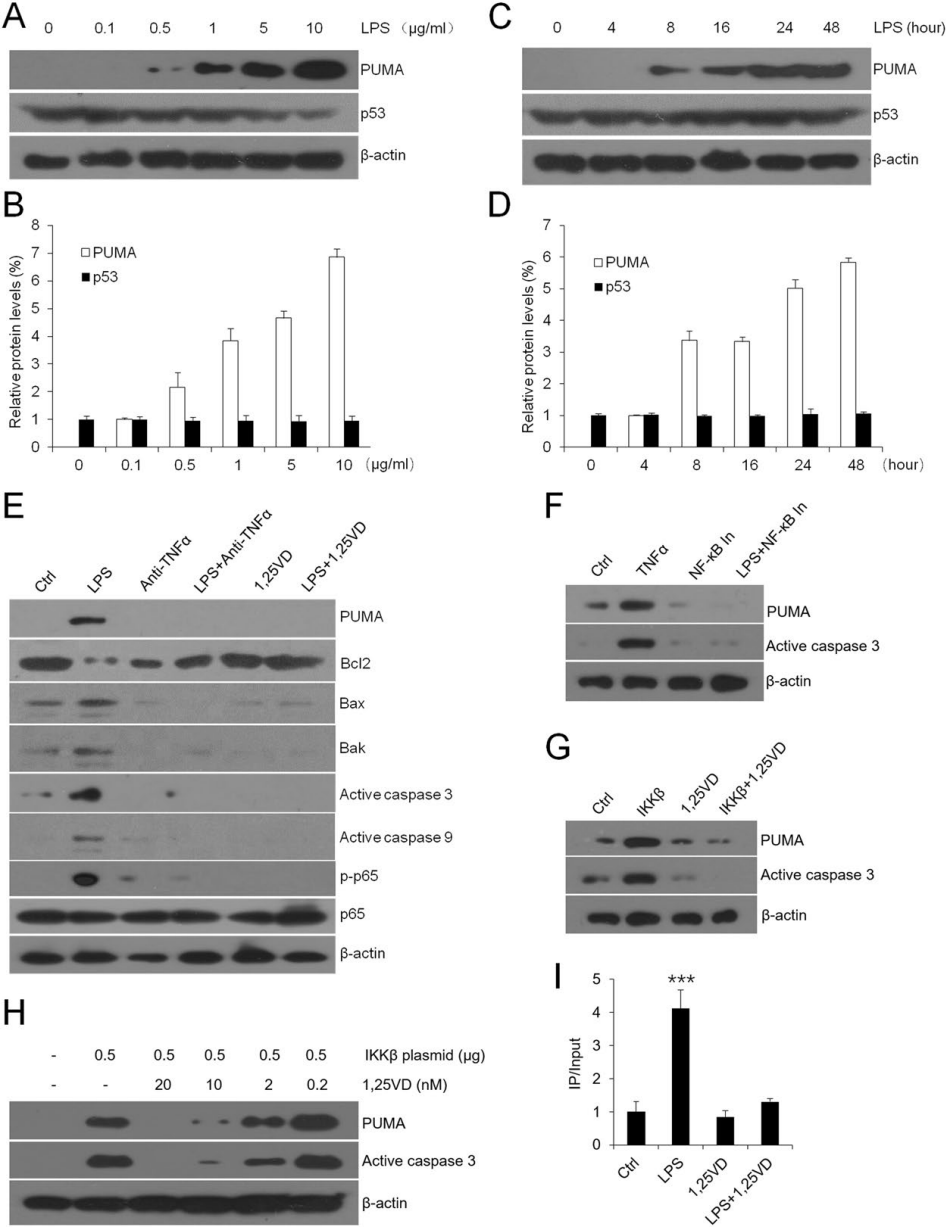
1,25(OH)_2_D_3_ suppresses LPS-induced apoptogenic factors in HaCat cells. (**A** and **B**) Expressions of PUMA and p53 with a concentration-dependent treatment of LPS tested by western blot (**A**) and quantified using densitometric analyses (**B**). (**C** and **D**) Expression of PUMA and p53 with a time-course treatment of LPS (10 μg/ml) tested by western blot (**C**) and quantified using densitometric analyses (**D**). (**E**) HaCat cells were pretreated with anti-TNFα (4 μg/ml), 1,25(OH)_2_D_3_ (20 nM) or vehicle for 12 hours, following 24-hour stimulations of LPS (10 µg/ml) as shown. (**F**) Western blot measurements of HaCat cells treated or untreated with TNFα (10 µg/ml) in the presence or absence of NF-κB inhibitor (20 nM). (**G**) HaCat cells were transfected with IKKβ-expressing plasmid or control vector after 12-hour treatment of 1,25(OH)_2_D_3_ as indicated. Western blot were used to analyze cell lysates. (H) HaCat cells were transfected with IKKβ-expressing plasmid, followed by 1,25(OH)_2_D_3_ treatments as indicated. Cell lysates were analyzed using western blot. (I) ChIP assays analyses of HaCat cells pretreated with 1,25(OH)_2_D_3_ or vehicle, and then stimulated by LPS or saline for 6 hours as shown, using anti-p65 antibodies, ***P < 0.001 vs. Ctrl. Ctrl, control; 1,25VD, 1,25(OH)_2_D_3_.

Increased epithelial cell apoptosis is thought to accelerate OLP development via damaging the mucosal barrier^14^. As known, PUMA is a key pro-apoptotic regulator^21^. In order to elucidate the function of LPS on cell apoptosis, we tested PUMA and p53 expressions in HaCat cells. Interestingly, protein activities of PUMA were significantly and largely up-regulated both in a LPS concentration-dependent manner (Fig. 4A and B) and in a LPS (10 µg/ml) time course-dependent way (Fig. 4C and D). However, the levels of p53 in HaCat cells were not induced by LPS (Fig. 4A–D). To address whether LPS modulates PUMA expression through TNFα-NF-κB signaling pathway, we carried out western blot experiments for detection. As shown, anti-TNFα neutralizing reagent successfully inhibited overexpressions of phosphorylated-NF-κB p65, PUMA, active caspase 3 and active caspase 9 which were induced by LPS (Fig. 4E). Importantly, LPS induced other pro-apoptotic factors (such as Bax and Bak) and suppressed the anti-apoptotic factor Bcl2, and these functions of LPS were blocked by anti-TNFα (Fig. 4E). Consistently, NF-κB inhibitor attenuated TNFα-stimulated apoptogenic proteins and, on the contrary, inhibitor of nuclear factor kappa-B kinase beta (IKKβ) transfection largely induced the levels of them (Fig. 4F and G). These results totally confirm that LPS plays its pro-apoptotic role strongly dependent on TNFα-NF-κB signaling pathway.

1,25(OH)_2_D_3_ completely overcome the activations of PUMA and other apoptosis-related factors after LPS treatment (Fig. 4E). Additionally, the inhibitory role of 1,25(OH)_2_D_3_ in apoptosis was subdued along with decreasing amounts of vitamin D in HaCat cells (Fig. 4H). In order to address the mechanism underling vitamin D’s mediation on LPS-induced apoptogenic factors, we performed ChIP assays. Here our data validated that 1,25(OH)_2_D_3_ stopped LPS-activated p65 from binding to its κB site in the promoter of *PUMA* gene in keratinocytes (Fig. 4I). These observations provide compelling evidence to explain the suppressive effect of 1,25(OH)_2_D_3_ on pro-apoptotic factors.

### Levels of PUMA are up-regulated strongly in the inflamed biopsies of human patient

**Figure 5:**
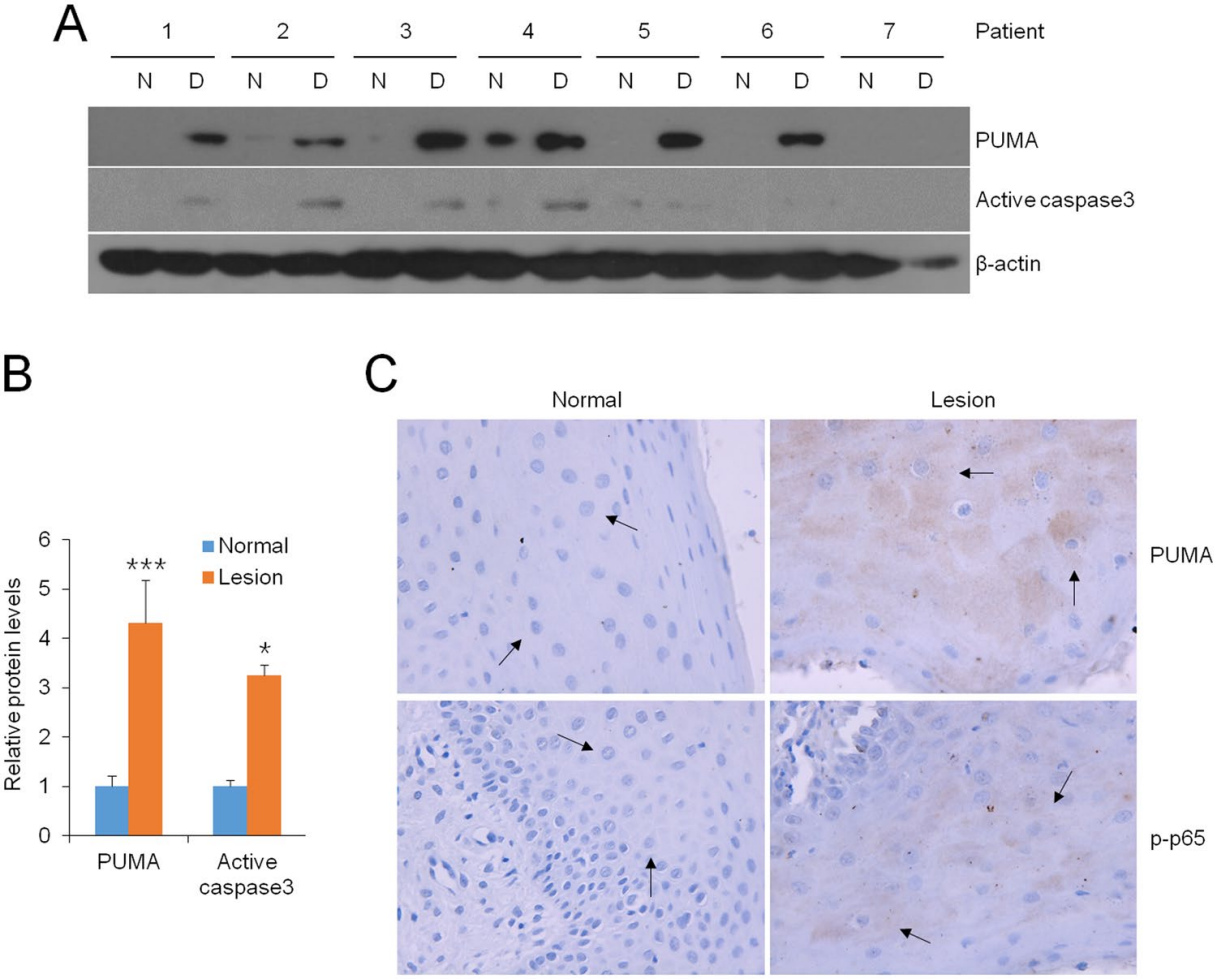
PUMA increase is strong in buccal mucosal biopsies of OLP patients. (**A**) Western blot measurements of lesion tissues (L) and adjacent normal biopsies (N) from OLP patients. (**B**) Relative protein levels of PUMA and active caspase3 in the diseased tissues in contrast to normal ones analyzed by densitometry, ***P < 0.001 vs. corresponding control, n = 14. (**C**) Immunostaining with anti-PUMA and anti-p-p65 antibodies in buccal mucosal biopsies, Magnification 400×.

To assess the expression of PUMA in OLP, we collected epithelium of oral mucosal for the following exploration. As shown in Fig. 5, PUMA expressions were enhanced significantly (>400%) and active caspase3 levels were also augmented largely (>300%) in the lesion, in contrast to the normal tissues (Fig. 5A and B). In accordant with this finding, immunostaining data showed that oral epithelial cells were robustly positive for PUMA and p-p65 expressions in the diseased samples (Fig. 5C), suggesting epithelial cells chiefly contributed the induction of PUMA and p-p65 in oral mucosa.

## Discussion

**Figure 6:**
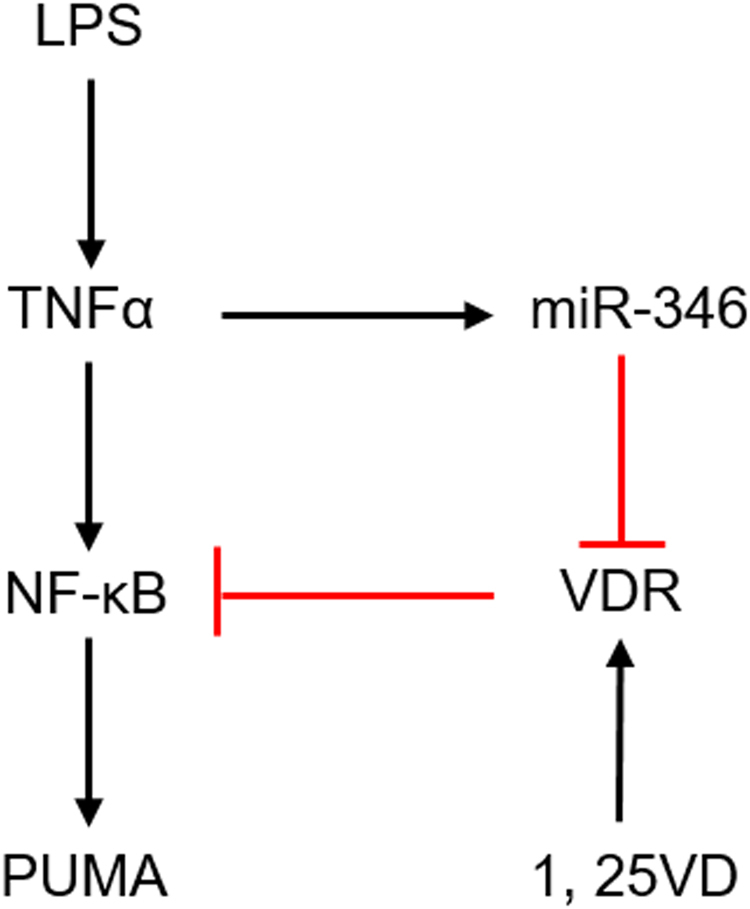
Schematic illustration of regulatory roles of 1,25(OH)_2_D_3_-VDR signaling in LPS-induced pro-apoptotic factor induction. 1,25VD, 1,25(OH)_2_D_3_.

OLP is a helper T-cell type 1 (Th1)-driven chronic inflammatory disorder which can be converted into cancer, and the average malignant transformation rate of it is approximate 1.09% as reported^27^. Albeit we have stated that vitamin D/VDR signaling pathway can relieves OLP development by mediating NF-κB pathway in previous study, the molecular mechanism of this regulatory process remains obscure. In this study, we demonstrate that LPS-induced VDR down-regulation enhances PUMA levels (Fig. 6), promoting epithelial cells apoptosis and impairing mucosal barrier.

The association between vitamin D/VDR and inflammatory diseases has been established for a long time^28,29^. The lack of vitamin D in patients with mucosal inflammation is common, even in the recovery course of diseases^30,31^. In experimental animal studies, VDR deficiency of colonic epithelial cells exacerbates T-cell transfer-induced or chemically-induced colitis^21^, revealing local VDR in epithelial layer protects mucosa against inflammation. In another work, vitamin D/VDR signaling is reported to attenuate LPS-induced lung injury through protecting pulmonary epithelial barrier^32^. Our previous investigations have confirmed that the expression of VDR in OLP biopsies is reduced compared with normal ones at the first time^20^. In agreement with this observation, the status of serum 25-hydroxyvitamin D of OLP patients is also lower than that of control. Since certain oral bacteria are thought to challenge mucosal barrier and involved in the onset of OLP, in the current work we used LPS-stimulated HaCat cells to seek the reason of VDR’s decrease in keratinocytes. Our data indicate that LPS robustly induces TNFα expression, conversely, the expression of VDR is reduced significantly. These findings are in accordant with other investigations reported early^32–34^. Critically, we note that LPS plays its suppressive role in VDR dependent on TNFα (Fig. 6). As previous observations have revealed that TNFα-induced miR-346 plays a down-regulated role in VDR in colon^24^, this time we further demonstrate miR-346 is able to reduce VDR expression in HaCat cells and the inhibitory function of TNFα is reversed by miR-346 inhibitor, suggesting VDR decrease in keratinocytes is caused by LPS-TNFα-miR-346 signaling pathway. Moreover, we confirm that LPS targets to the 3′UTR sequence of VDR transcript to stop its expression (Fig. 2), which is consistent with previous studies^24^.

We have indicated that Th-1 related cytokines levels of oral diseased mucosa in OLP are largely promoted in recent findings. In this report, we validate our early observation concerning mucosal cytokines expression in a new cohort of OLP patients. Compellingly, TNFα expression of oral mucosal epithelia derived from these individuals are remarkably elevated, while VDR status in these epithelial cells is restrained. The human data of this time have better explained that the VDR reduction, in oral mucosal epithelial cells, is closely related with immunologic reactions of inflamed tissues. Indeed, the decrease of VDR is due to TNFα-induced miR346 mediation, but the source of this kind of cytokine attracts numerous attentions. Undoubtedly, TNFα activity can be enhanced by its upstream stimulator (LPS) in epithelium, which is the key topic of this study. However, to some extent, TNFα is also able to be secreted by CD4^+^ T cells in the lamina propria. Therefore, how TNFα from subepithelial layer affects VDR in keratinocytes are attractive and requires further explorations.

The mucosal barrier, which consists of epithelial cells and the connected structure between them, prevents soft tissues against microbes’ invasion. In diseased tissues from patients with OLP, bacteria can be found both in ulcerated areas and in non-ulcerated regions^12^, implying epithelial barrier impairment. Apoptosis, a programmed cell death mechanism, is believed to compromise epithelial barrier in tremendous investigations^11,14^. To explore the impact of microbes on keratinocytes apoptosis, we tested the expression of PUMA with LPS treatment. As shown in this study, LPS improves the activities of PUMA significantly, suggesting bacteria accelerate apoptosis development of epithelia. Since previous studies have reported that epithelial VDR maintains mucosal homeostasis^35^, in this case, LPS-induced VDR reduction might be involved in epithelia apoptosis. Here, we manifest that 1,25(OH)_2_D_3_ abrogates LPS-stimulated PUMA and active caspase 3 inductions. Moreover, we embarked on explorations to dissect the molecular basis underlying 1,25(OH)_2_D_3_ blockade associated with apoptosis-related factors expressions. In HaCat cells, 1,25(OH)_2_D_3_ inhibits IKKβ-induced PUMA, and this inhibition can be reversed by decreasing amount of 1,25(OH)_2_D_3_ treatment in turn. Additionally, 1,25(OH)_2_D_3_ blocks LPS-induced p65 binding to the κB site located in *PUMA* promoter. The two compelling evidences support the view that 1,25(OH)_2_D_3_ regulation of apoptosis is due to the impediment of NF-κB activation (Fig. 6), and this conclusion is consistent with early studies. Importantly, PUMA signals are enhanced in the epithelium of inflamed biopsies from the same new cohort of patients mentioned above, suggesting apoptosis action is robust in keratinocytes of OLP tissues. Furthermore, we found that LPS takes effect on other apoptosis factors (such as Bcl2, Bax and Bak) dependent on TNFα in keratinocytes, and this finding requires more explorations. Taken together, it is well understood that LPS-induced VDR decrease in keratinocytes enhances apoptosis, leading to mucosal barrier disruption and OLP occurrence. Although some studies have demonstrated that epithelial barrier damage caused by bacteria is the pathogenesis of OLP^12^, we further elaborate the molecular mechanism whereby LPS disrupts mucosal epithelium and detail the function of vitamin D/VDR involved in this process creatively.

In conclusion, we provide convictive evidence that LPS down-regulates VDR expression in oral mucosal epithelia dependent on TNFα-miR346 signaling, and we further suggest that vitamin D/VDR can suppress LPS-induced keratinocytes apoptosis by regulating NF-κB pathway. Thus, vitamin D/VDR plays a protective role in the integrity of oral mucosal barrier to overcome the challenge of bacteria, stopping or delaying OLP development. Given the relatively insufficient VDR levels detected in OLP patients, targeting VDR expression in oral epithelial cells might be a helpful approach for the management of OLP.
